# A Phase II Study of Flavopiridol in Patients With Previously Untreated Advanced Soft Tissue Sarcoma

**DOI:** 10.1155/SRCM/2006/64374

**Published:** 2006-10-01

**Authors:** Don G. Morris, Vivien H. C. Bramwell, Robert Turcotte, Alvaro T. Figueredo, Martin E. Blackstein, Shail Verma, Sarah Matthews, Elizabeth A. Eisenhauer

**Affiliations:** ^1^Department of Medicine, Tom Baker Cancer Centre, University of Calgary, Alberta, Canada T2N 4N2; ^2^Department of Orthopaedic Surgery, McGill University Health Centre, Montreal, Quebec, Canada H3G 1A4; ^3^Department of Medical Oncology, Juravinski Cancer Centre, Hamilton Health Scineces, Hamilton, Ontario, Canada L8V 5C2; ^4^Department of Anat. (Histol) & Med, Mount Sinai Hospital, University of Toronto, Toronto, Ontario, Canada M5G 1X5; ^5^Department of Medicine, University of Ottawa, Ottawa, Ontario, Canada K1H 1C4; ^6^NCIC Clinical Trials Group, Queen's University, Kingston, Ontario, Canada K7L 3N6

## Abstract

*Purpose*. Flavopiridol is a potent cyclin-dependent kinase (CDK) inhibitor that has preclinical activity in many tumours. This synthetic flavonoid was tested in a phase II nonrandomized, nonblinded multicentre clinical trial to determine its activity and toxicity in patients with previously untreated metastatic or locally advanced soft tissue sarcoma. *Methods*. A total of 18 patients with histologically confirmed nonoperable soft tissue was treated with flavopiridol administered at a dose of 50 mg/*m* ^2^ IV over 1 hour daily ×3 days every 3 weeks. *Results*. Eighteen patients were accrued to the study over a period of 6 months. No objective responses were noted in the seventeen evaluable patients. Eight patients (47%) exhibited stable disease after 2 cycles (median duration of 4.3 months (range 1.4–6.9 months). Kaplan-Meier estimates for 3- and 6-month progression-free survivial rates were 44 percent and 22 percent, respectively. The only grade 3 toxicities were diarrhea (*N* = 2), nausea (*N* = 2), gastritis (*N* = 1), and fatigue (*N* = 1). Ninety-four percent of patients received ≥ 90% of the planned dose intensity, during 55 treatment cycles. *Conclusions*. Flavopiridol was well tolerated at the dose and schedule used in this study, however, no objective treatment responses were seen and thus our results do not support further exploration of flavopiridol as a monotherapy at this dose and schedule in soft tissue sarcomas.

## INTRODUCTION

Soft tissue sarcomas (STS) constitute a heterogenous group of cancers that all share origins from mesenchymal tissue. The annual incidence of STS is approximately 9 420 cases per year in the United States, resulting in 3 490 deaths per year and represents approximately 1% of all adult malignancies [1]. Even with current advances in treatment, mortality rates still approach 50 percent for newly diagnosed patients. Current treatments for nonoperable or metastatic STS remain unsatisfactory. Although, there are multiple drugs that have modest efficacy in treating STS, the single most effective drug is doxorubicin (75 mg/m^2^) with response rates generally less than 25% in recent studies [2]. The use of combination chemotherapy, particularly ifosfamide/doxorubicin-based regimens, has increased response rates, however, there is still no significant improvement in survival and significantly increased toxicity has been found [3, 4]. Further, despite promising results in phase II studies, randomized phase III trials evaluating dose escalation of doxorubicin/ifosfamide-based regimes supported by growth factors have not shown a survival benefit [5, 6]. Given these poor results, there has been a trend towards the use of single agent chemotherapy in patients receiving palliative treatment.

Newer agents with different/novel mechanisms of action are desperately needed. Preclinical investigations into the role of cyclins and cyclin-dependent kinases (CDKs) in sarcoma indicate that many STSs acquire changes that disrupt checkpoint control (ie, CDK overexpression) resulting in unregulated progression through the cell cycle. Alterations in the expression of cyclins D1,2,3, *E*, and *A* and the CDK inhibitors, p21 and p27 have all been shown to be markers of poor prognosis in many STS [7–10].

Flavopiridol, an N-methylpiperidinyl, chlorophenyl flavone, is an analogue of a naturally occurring flavonoid isolated from the bark of *Dysoxylum binectariferum*, a plant indigenous to India. It was the first potent CDK inhibitor to enter human clinical trials and inhibits the kinase activity of multiple CDKs with CDK 1,2,4 and 7 IC_50_s in the 100–400 nM range. It also interferes with the phosphorylation events necessary to activate CDKs and has been shown to downregulate transcription of the cyclin D1 gene [11] and vascular endothelial growth factor expression [12]. Flavopiridol xenograft model systems in nonsmall cell lung, breast, prostate, and several other tumours have shown cytotoxic activity [13, 14]. Several phase I clinical trials have established dose schedules that are well tolerated and achieve serum levels adequate for CDK inhibition. Published phase II studies in renal [15], gastric [16], nonsmall cell lung [17], and head and neck [18] cancers have used a continuous infusional schedule, however, in xenograft models, peak concentrations achieved in a bolus schedule correlated best with antitumour activity [19]. A phase I trial using a bolus, daily ×3 schedule in advanced neoplasms was associated with stabilization of disease in the setting of progressive disease prior to entry onto the trial [20]. A recent phase II study using flavopiridol (50 mg/m^2^) IV bolus daily ×3 in advanced renal cell carcinoma revealed an acceptable toxicity profile, an overall response rate of 12% and a stable disease rate of 41% [21]. Due to the ease of administration, evidence of clinical activity in other histologies and phase I/II experience, the daily ×3 schedule was adopted in this current study.

## METHODS

### Patient eligibility

Patients with documented metastatic or locally advanced soft tissue sarcoma, not curable by other means, were eligible for this trial. Eligibility criteria were as follows: histologically documented soft tissue sarcoma; presence of at least one site of unidimensionally measurable clinically and/or radiologically documented disease; age ≥ 18 years, life expectancy of at least 12 weeks; Eastern Cooperative Oncology Group performance status ≤ 2; and no prior systemic therapy for metastatic disease. Prior adjuvant chemotherapy was permitted as long as treatment had been completed ≥ 6 months since the last dose of therapy. Patients could not have received radiotherapy to the sole site of measurable disease unless it was a current site of progressive disease, and no more than 25% of functioning bone marrow could have been irradiated. Previous surgery was allowed if ≥ 4 weeks prior to initiating treatment. Laboratory requirements included the following: absolute granulocyte count ≥ 1.5 × 10^9^/L; platelet count ≥ 100×10^9^/L; AST ≤ 2.5× upper normal limit (UNL); serum creatinine ≤ UNL; bilirubin ≤ UNL. All patients must have given informed consent according to the requirements of their local Institutional and/or University Human Experimentation Committee.

### Treatment

Flavopiridol (Aventis Pharmaceuticals, Inc) was supplied by the Cancer Therapy Evaluation Program (CTEP), Division of Cancer Treatment and Diagnosis, National Cancer Institute, Bethesda, Md, USA. The flavopiridol was supplied as a sterile 10 mg/ml solution in vials of 50 mg of free base equivalent. The individually calculated dose was diluted prior to infusion with 0.9% sodium chloride injection USP or 5% dextrose injection USP to final concentrations ranging from 0.09 to 0.5 mg/ml flavopiridol.

Flavopiridol was administered in the outpatient setting as a 1 hour infusion at a dose of 50 mg/m^2^ daily ×3 days every 21 days. Vital signs were monitored every 30 minutes ×5 beginning at the start of infusion until 1 hour post infusion. Antidiarrheal prophylaxis and antiemetic prophylaxis were prescribed as follows: 4 chewable Pepto Bismol tablets 1 hour before first dose of flavopiridol, then 2 tablets every 6 hours until 12 hours after the last dose of flavopiridol (day 3), ondansetron 8 mg orally every 12 hours beginning 12 hours before treatment and continuing until 12 hours after the last dose of flavopiridol (day 3). Loperamide was started if diarrhea occurred, using a dose regimen of 2 mg orally q.2 h while awake and q.4 h during sleep until 12 h diarrhea-free. Dose reductions were allowed (dose level: 1 = 37.5 mg/m^2^/d, dose level: 2 = 28 mg/m^2^/d), if the following toxicities were seen: diarrhea ≥ grade 3 associated with mucus or dehydration; nausea and vomiting ≥ grade 3 despite antiemetics; granulocyte nadir < 0.5×10^9^/L and/or neutropenia with fever or infection; platelet nadir < 25 × 10^9^/L and/or thrombocytopenic bleeding; or any nonhematologic toxicity (except alopecia) ≥ grade 3. Patients requiring more than two dose reductions were removed from protocol treatment.

Treatment continued until progression, or for 2 cycles after complete or stable partial response. Nonresponding stable patients continued on treatment until progression, or alternatively could be removed from therapy after 6 cycles at the investigator's discretion. After termination from protocol treatment, all patients were seen at 4 weeks, then every 3 months until progression or death.

### Response and toxicity assessment

Patients were considered evaluable for response if they had received at least 1 cycle of therapy and had their disease reevaluated. Patients were evaluated for response every 6 weeks (every 2 cycles) using the same investigations that demonstrated measurable disease at baseline. Response and progression were evaluated using the RECIST criteria (response evaluation criteria in solid tumors) [22].

Patients were considered evaluable for toxicity after their first infusion of flavopiridol. A history and physical examination were performed on day 1 of each cycle with an assessment of toxicity during the previous treatment interval. CBC, platelets, differential, bilirubin and AST were performed on days 1, 8, and 15, and BUN, creatinine, electrolytes, LDH, fasting glucose, and alkaline phosphatase were performed on day 1 of each cycle. Toxicities were graded according to the NCI Common Toxicity Criteria Version 2.0 [23].

### Statistical analysis

The primary endpoint of this phase II study was objective response rate, with secondary endpoints of toxicity, time to progression, early progression rate, and response duration. In order to minimize the expected number of patients treated in the event that the regimen proved to be very disappointing or very successful, a two-stage design was used for patient accrual. This design tests the null hypothesis (H_0_) that the true response rate is < 5% versus the alternative hypothesis (H_A_) that the true response rate is > 20%. The significance level (ie, the probability of rejecting H_0_ when it is true) is 0.058. The power is 0.865 when the true response probability is 20%. If there were no responses after the first 15 patients, the response rate is concluded to be < 5% and accrual was to be stopped. If there were one or more responses, accrual was to continue to 30 evaluable patients.

## RESULTS

### Patient characteristics

A total of 18 patients were accrued onto this study. All patients were evaluable for toxicity and 17 patients were evaluable for response (1 patient discontinued protocol without disease assessment). The characteristics of the patients are outlined in Table 1. Four patients had received treatment in the adjuvant setting (3 chemotherapy, 1 immunotherapy) and 10 had received radiation therapy prior to entry onto the trial. All patients were stage IV at the time of entry onto the trial. The most common sites of metastatic disease were pulmonary (13/18), hepatic (5/18) and subcutaneous (5/18). The histological subtypes are listed in Table 2.

Overall, flavopiridol was well tolerated with 17/18 patients receiving the planned dose intensity. One patient had a protocol mandated dose reduction due to grade 4 neutropenia (a second patient who experienced grade 4 neutropenia failed to receive the dose reduction). Two doses delays occurred during the trial, both nontoxicity related. The toxicities reported to be possibly related to the protocol treatment are outlined in Table 3. The most common toxicities included diarrhea (83%), nausea (67%), fatigue (61%), anorexia (50%), and vomiting (39%). The toxicities were generally grade 1 or 2, although two patients experienced grade 3 diarrhea and one patient was hospitalized for gastritis while neutropenic. Three patients experienced grade 3/4 leucopenia and 6 patients with grade 3/4 granulocytopenia (see Table 4). The median time to leukocyte and granulocyte nadir was 8-9 days. Biochemical toxicities (see Table 5) were generally mild with one patient exhibiting grade 2 total bilirubin and alkaline phosphatase elevations.

### Treatment and response

Ninety-four percent of patients received ≥ 90% of planned dose intensity. A total of 55 cycles of treatment were given. The median number of treatment cycles was 3 (range 1–6 cycles). One patient had a dose reduction due to treatment toxicity, and 2 patients had dose delays due to nontreatment related factors. Of the 18 patients entered onto the study, 14 patients were removed from the study for progressive disease (4/14 symptomatic progression). There was one tumour related death. Of the three remaining patients one completed the study and a further two patients were removed from the study upon the advice of their physician.

The rates of complete and partial response, stable disease and progressive disease are given in Table 6. Of the seventeen patients evaluable for response (one patient discontinued treatment without tumour assessment), there were no documented complete or partial responses (overall response rate = 0.0%, 95% CI: 0.0–16.2%). As per the two-stage design of the study, accrual was thus discontinued after the initial cohort of patients was enrolled. Stable disease for more than 2 cycles was documented in eight patients (47%) all with different histologies, with a median duration of 4.3 months (range 1.4–6.9 months). Interesting, estimates of progression free survival at 3 months and 6 months were 44% and 22%, respectively suggestive of possible disease stabilization.

## DISCUSSION

Flavopiridol was the first example of a cyclin-dependent kinase inhibitor to be tested in clinical trials. Although it exhibits a relative selectivity for CDKs, it also has been reported to inhibit protein kinase C at an IC_50_ of 6 umol/L, cyclic adenosine triphosphate-dependent kinase and epidermal growth factor-receptor kinase at IC_50_s of 145 and 25 umol/L, respectively [24]. Flavopiridol has also been reported to suppress the transcription of cell-cycle specific genes, including cyclin D1 and induce apoptosis in preclinical models at micromolar concentrations [11, 13, 25–27]. Multiple phase I and II trials have investigated the dose/schedule, toxicity and efficacy of flavopiridol. Tan et al reported on a phase I trial of a daily 1 hour infusion schedule that achieved the minimal inhibitory concentrations (> 3 umol) that inhibited CDKs in vitro [20]. There was a suggestion from this study that the toxicity profile found with the 72 hour infusion schedule was altered with bolus scheduling, that is, decreased thrombotic and asthenic complications and increased diarrhea/myelosuppression that correlates with peak plasma concentrations, consistent with our study.

This study demonstrates that flavopiridol can be given safely in an outpatient setting using a bolus dose schedule. Expected toxicities of diarrhea, nausea, and vomiting were manageable as long as patients received adequate prophylactic treatment. Of the 18 patients enrolled on the study, only one patient required a dose reduction. Hematologic toxicity was modest.

Although there were no objective complete or partial responses seen in our patient population, 47% of evaluable patients experienced stable disease for at least 2 cycles, the majority of whom had progressive disease at study entry.

Despite the potential for activity in STS, no objective responses were seen. It has been postulated that flavopiridol may act as a modulator of cytotoxic chemotherapeutic agents, such as mitomycin C in gastric cancer, the taxanes, anthracyclines, gemcitabine in breast cancer and ara-c in acute leukemias [25, 28–30].

Resistance to flavopiridol associated with overexpression of ATP-binding proteins and cyclin E has been described in breast and colon cancer cell lines, respectively [31, 32]. There has also been a report of an interaction between flavopiridol and MRPI, suggesting a potential drug resistance plenotype [33].

In some recent clinical trials, stable disease has been added to objective response and termed “clinical benefit response” [34]. This study was not designed to assess “clinical benefit response” or to address the possibility of cytostatic mechanisms of action; therefore accrual was discontinued after no responses were seen in the first seventeen evaluable patients, as required by the statistical design of the study. An assessment of “clinical benefit response” may be an important component in the design of future studies particularly for agents that are postulated to be cytostatic in action. Further, Van Glabbeke et al have suggested that 6 month progression free survival rates of > 30% in phase II trials may provide evidence of activity in first line soft tissue sarcoma to carry agents into phase III trials [35]. Our results of 3 month and 6 month PFS rates of 44% and 22%, respectively, however, do not compare favorably to these numbers. If there is a role of flavopiridol in STS as a treatment modality, it may well be in the setting of combination with conventional cytotoxic chemotherapy.

## Figures and Tables

**Table 1:** Patient characteristics (*n* = 18 patients).

Number of patients	

Patients entered on study	18
Evaluable for toxicity	18
Evaluable for response	17
Median age (range)	52(36–79)

Gender	

Female	6
Male	12

Performance status	

0	7
1	9
2	2

Prior therapy	

Adjuvant chemotherapy	3
Adjuvant immunotherapy	1
Radiotherapy	10

Number of Prior chemo regimens	

0	15
1	3

Sites of disease	

Abdomen	1
Adrenal	1
Bone	3
Chest wall	1
Kidney	1
Liver	5
Lung	13
Bone marrow	1
Nodes	4
Pelvis	3
Pleural effusion	1
Pleura	1
Retroperitoneal	2
Subcutaneous soft tissue	5

Number of sites of disease	

1	4
2	6
3	7
≥ 4	1

Stage IV disease	18

**Table 2:** Histology (*n* = 18 patients).

	
Number of patients	

Histology	
Angiosarcoma	2
Clear cell	1
Epithelial sarcoma	1
Fibrosarcoma	1
GI stromal cell	2
Malignant hemangiopericytoma	1
Leiomyosarcoma	2
Liposarcoma	2
Malignant fibrous histiocytoma	1
Malignant schwannoma	1
Synovial sarcoma	1
Sarcoma NOS	3

**Table 3:** Treatment-related^*^ nonhematologic toxicities (worst by patient) (*n* = 18 patients).

Grade^**^							

Toxicity	1	2	3	4	5	Total	%pts

Flu-like symptoms							
Fever	1	—	—	—	—	1	(5.6)
Fatigue	5	5	1	—	—	11	(61.1)
Rigors, chills	4	—	—	—	—	4	(22.2)
Gastrointestinal							
Anorexia	6	3	—	—	—	9	(50.0)
Constipation	—	2	—	—	—	2	(11.1)
Diarrhea	7	6	2	—	—	15	(83.3)
Dysphagia	2	—	—	—	—	2	(11.1)
Mouth dryness	1	—	—	—	—	1	(5.6)
Gastritis	—	—	1	—	—	1	(5.6)
Nausea	6	4	2	—	—	12	(66.7)
Salivary gland changes	2	—	—	—	—	2	(11.1)
Stomatitis	1	—	—	—	—	1	(5.6)
Taste disturbance	6	—	—	—	—	6	(33.3)
Vomiting	4	3	—	—	—	7	(38.9)
Hemorrhage							
Melena/GI bleeding	1	—	—	—	—	1	(5.6)
Hematuria	—	1	—	—	—	1	(5.6)
Infection							
Infection w/o neutropen	—	1	—	—	—	1	(5.6)
Pain							
Abdominal pain	1	1	—	—	—	2	(11.1)
Headache	1	1	—	—	—	2	(11.1)
Myalgia	2	—	—	—	—	2	(11.1)
Dermatology							
Alopecia	3	—	—	—	—	3	(16.7)
Dry skin	—	1	—	—	—	1	(5.6)
Injection site reaction	1	—	—	—	—	1	(5.6)
Rash/desquamation	1	—	—	—	—	1	(5.6)
Any	17	14	6	0	0	18	(100.0)

*Considered by investigator to be “possibly” or “definitely” related to protocol treatment.

**Toxicity graded according to NCI Common Toxicity Criteria Version 2.0.

**Table 4:** Treatment-related hematologic toxicity (worst by patient).

	No of evaluable^*^ patients	Grade^**^				

	—	0	1	2	3	4
Granulocytes	18	6	2	4	4	2
Hemoglobin	18	7	8	2	1	—
Platelets	18	14	4	—	—	—
WBC	18	8	4	3	2	1

*Includes patients with at least one evaluable cycle (blood count done between days 7–16).

**Toxicity graded according to NCI Common Toxicity Criteria Version 2.0.

**Table 5:** Treatment-related biochemical toxicity (worst by patient)^*^.

Toxicity	No of evaluable^*^	Grade^**^				
	patients					

	—	0	1	2	3	4
Creatinine	18	17	1	—	—	—
SGOT (AST)	15	17	3	—	—	—

Bilirubin						

All patients	16	15	—	1	—	—
Normal baseline	15	14	—	1	—	—

Alkaline phosphatase						

All patients	15	13	1	1	—	—
Normal baseline	14	13	1	—	—	—

Hyperglycemia						

All patients	13	8	5	—	—	—
Normal baseline	8	6	2	—	—	—

*Indicates patients with at least one blood sample taken after day 1.

**Toxicity graded according to the NCI Common Toxicity Criteria Version 2.0.

**Table 6:** Confirmed response (*n* = 17 evaluable patients). Overall response rate—0.0% (95% CI: 0.0–16.2%).

			Duration (months)	

		No patients	Median	Range
Complete response	(CR)	0	—	—
Partial response	(PR)	0	—	—
Stable disease	(SD)	8	4.3	1.4–6.9
Progressive disease	(PD)	9	—	—

## References

[1] Jemal A, Murray T, Ward E (2005). Cancer statistics, 2005. *CA-A Cancer Journal for Clinicians*.

[2] Verweij J, Pinedo HM, Pinedo HM, Verweij J, Suti HD (1991). Systemic treatment of advanced or metastatic soft tissue sarcoma. *Soft Tissue Sarcomas: New Developments in the Multidisciplinary Approach to Treatment*.

[3] Edmonson JH, Ryan LM, Blum RH (1993). Randomized comparison of doxorubicin alone versus ifosfamide plus doxorubicin or mitomycin, doxorubicin, and cisplatin against advanced soft tissue sarcomas. *Journal of Clinical Oncology*.

[4] Blum RH, Edmonson JH (1995). Investigations of ifosfamide for adult soft tissue sarcoma in ECOG. *European Journal of Cancer*.

[5] Le Cesne A, Judson I, Crowther D (2000). Randomized phase III study comparing conventional-dose doxorubicin plus ifosfamide versus high-dose doxorubicin plus infosfamide plus recombinant human granulocyte-macrophage colony-stimulating factor in advanced soft tissue sarcomas: a trial of the european organization for research and treatment of cancer/soft tissue and bone sarcoma group. *Journal of Clinical Oncology*.

[6] Bui NB, DeMaille MC, Chevreau C (1998). _9_MAID vs MAID + 25% with G-CSF in adults with advanced soft tissue sarcoma (STS). First results of a randomized study of the FNCLCC Sarcoma Group. *Proceedings of the American Society for Clinical Oncology*.

[7] Kim SH, Lewis JJ, Brennan MF, Woodruff JM, Dudas M, Cordon-Cardo C (1998). Overexpression of cyclin D1 is associated with poor prognosis in extremity soft-tissue sarcomas. *Clinical Cancer Research*.

[8] Huuhtanen RL, Blomqvist CP, Böhling TO (1999). Expression of cyclin A in soft tissue sarcomas correlates with tumor aggressiveness. *Cancer Research*.

[9] Pindzola JA, Palazzo JP, Kovatich AJ, Tuma B, Nobel M (1998). Expression of p21(WAF1/CIP1) in soft tissue sarcomas: a comparative immunohistochemical study with p53 and Ki-67. *Pathology Research and Practice*.

[10] Kawauchi S, Goto Y, Liu XP (2001). Low expression of p27^kip1^, a cyclin-dependent kinase inhibitor, is a marker of poor prognosis in synovial sarcoma. *Cancer*.

[11] Carlson B, Lahusen T, Singh S (1999). Down-regulation of cyclin D1 by transcriptional repression in MCF-7 human breast carcinoma cells induced by flavopiridol. *Cancer Research*.

[12] Melillo G, Sausville EA, Cloud K, Lahusen T, Varesio L, Senderowicz AM (1999). Flavopiridol, a protein kinase inhibitor, down-regulates hypoxic induction of vascular endothelial growth factor expression in human monocytes. *Cancer Research*.

[13] Shapiro GI, Koestner DA, Matranga CB, Rollins BJ (1999). Flavopiridol induces cell cycle arrest and p53-independent apoptosis in non-small cell lung cancer cell lines. *Clinical Cancer Research*.

[14] (1998). *Investigator's Brochure, HMR 1275*.

[15] Stadler WM, Vogelzang NJ, Amato R (2000). Flavopiridol, a novel cyclin-dependent kinase inhibitor, in metastatic renal cancer: a University of Chicago phase II consortium study. *Journal of Clinical Oncology*.

[16] Schwartz GK, Ilson D, Saltz L (2001). Phase II study of the cyclin-dependent kinase inhibitor flavopiridol administered to patients with advanced gastric carcinoma. *Journal of Clinical Oncology*.

[17] Shapiro GI, Supko JG, Patterson A (2001). A phase II trial of the cyclin-dependent kinase inhibitor flavopiridol in patients with previously untreated stage IV non-small cell lung cancer. *Clinical Cancer Research*.

[18] Patel V, Senderowicz AM, Pinto D Jr (1998). Flavopiridol, a novel cyclin-dependent kinase inhibitor, suppresses the growth of head and neck squamous cell carcinomas by inducing apoptosis. *Journal of Clinical Investigation*.

[19] Arguello F, Alexander M, Sterry JA (1998). Flavopiridol induces apoptosis of normal lymphoid cells, causes immunosuppression, and has potent antitumor activity in vivo against human leukemia and lymphoma xenografts. *Blood*.

[20] Tan AR, Headlee D, Messmann R (2002). Phase I clinical and pharmacokinetic study of flavopiridol administered as a daily 1-hour infusion in patients with advanced neoplasms. *Journal of Clinical Oncology*.

[21] Van Veldhuizen PJ, Faulkner JR, Lara PN Jr (2005). A phase II study of flavopiridol in patients with advanced renal cell carcinoma: results of Southwest Oncology Group Trial 0109. *Cancer Chemotherapy and Pharmacology*.

[22] Therasse P, Arbuck SG, Eisenhauer EA (2000). New guidelines to evaluate the response to treatment in solid tumors (RECIST guidelines). *Journal of the National Cancer Institute*.

[23] http://ctep.cancer.gov/forms/.

[24] Senderowicz AM (1999). Flavopiridol: the first cyclin-dependent kinase inhibitor in human clinical trials. *Investigational New Drugs*.

[25] Motwani M, Delohery TM, Schwartz GK (1999). Sequential dependent enhancement of caspase activation and apoptosis by flavopiridol on paclitaxel-treated human gastric and breast cancer cells. *Clinical Cancer Research*.

[26] Schrump DS, Matthews W, Chen GA, Mixon A, Altorki NK (1998). Flavopiridol mediates cell cycle arrest and apoptosis in esophageal cancer cells. *Clinical Cancer Research*.

[27] Wittmann S, Bali P, Donapaty S (2003). Flavopiridol down-regulates antiapoptotic proteins and sensitizes human breast cancer cells to epothilone B-induced apoptosis. *Cancer Research*.

[28] Bible KC, Kaufmann SH (1997). Cytotoxic synergy between flavopiridol (NSC 649890, L86-8275) and various antineoplastic agents: the importance of sequence of administration. *Cancer Research*.

[29] Schwartz GK, O'Reilly E, Ilson D (2002). Phase I study of the cyclin-dependent kinase inhibitor flavopiridol in combination with paclitaxel in patients with advanced solid tumors. *Journal of Clinical Oncology*.

[30] Ali S, El-Rayes BF, Aranha O, Sarkar FH, Philip PA (2005). Sequence dependent potentiation of gemcitabine by flavopiridol in human breast cancer cells. *Breast Cancer Research and Treatment*.

[31] Robey RW, Medina-Pérez WY, Nishiyama K (2001). Overexpression of the ATP-binding cassette half-transporter, ABCG2 (MXR/BCRP/ABCP1), in flavopiridol-resistant human breast cancer cells. *Clinical Cancer Research*.

[32] Smith V, Raynaud F, Workman P, Kelland LR (2001). Characterization of a human colorectal carcinoma cell line with acquired resistance to flavopiridol. *Molecular Pharmacology*.

[33] Hooijberg JH, Broxterman HJ, Scheffer GL (1999). Potent interaction of flavopiridol with MRP1. *British Journal of Cancer*.

[34] Merimsky O, Meller I, Flusser G (2000). Gemcitabine in soft tissue or bone sarcoma resistant to standard chemotherapy: a phase II study. *Cancer Chemotherapy and Pharmacology*.

[35] Van Glabbeke M, Verweij J, Judson I, Nielsen OS, on behalf of the EORTC Soft Tissue and Bone Sarcoma Group (2002). Progression-free rate as the principal end-point for phase II trials in soft-tissue sarcomas. *European Journal of Cancer*.

